# Marked Response to Nivolumab by a Patient With SMARCA4‐Deficient Undifferentiated Urothelial Carcinoma Showing High PD‐L1 Expression: A Case Report

**DOI:** 10.1002/cnr2.2127

**Published:** 2024-06-24

**Authors:** Yohei Arihara, Ginji Omori, Ko Kobayashi, Shintaro Sugita, Kazuyuki Murase, Tomohiro Kubo, Masashi Idogawa, Tadashi Hasegawa, Kohichi Takada

**Affiliations:** ^1^ Department of Medical Oncology Sapporo Medical University School of Medicine Sapporo Japan; ^2^ Department of Urology Sapporo Medical University School of Medicine Sapporo Japan; ^3^ Department of Surgical Pathology Sapporo Medical University School of Medicine Sapporo Japan; ^4^ Department of Medical Genome Sciences, Cancer Research Institute Sapporo Medical University School of Medicine Sapporo Japan

**Keywords:** carcinoma of unknown primary, immune checkpoint inhibitor, nivolumab, SMARCA4

## Abstract

**Background:**

*SMARCA4* is a component gene of the SWI/SNF (SWItch/Sucrose NonFermentable) chromatin remodeling complex; undifferentiated tumors associated with its functional deletion have been described in several organs. However, no established treatment for these tumors currently exists.

**Case:**

In this study, we report a case of a SMARCA4‐deficient undifferentiated urothelial carcinoma with high PD‐L1 expression that was effectively treated with nivolumab after early relapse following treatment for non‐invasive bladder cancer. The histological morphology of the rhabdoid‐like undifferentiated tumor of unknown primary led us to suspect a SWI/SNF‐deficient tumor, and subsequent immunostaining led to the diagnosis of a SMARCA4‐deficient undifferentiated tumor. This effort also led to the identification of the developmental origin of this SMARCA4‐deficient undifferentiated tumor as a non‐invasive bladder cancer. We also carried out a detailed immune phenotypic assay on peripheral T cells. In brief, a phenotypic change of CD8+T cells from naive to terminally differentiated effector memory cells was observed.

**Conclusion:**

Regardless of the organ of cancer origin or cancer type, SWI/SNF‐deficient tumors should be suspected in undifferentiated and dedifferentiated tumors, and immune checkpoint inhibitors may be considered as a promising treatment option for this type of tumor. The pathogenesis of SMARCA4‐deficient anaplastic tumors awaits further elucidation for therapeutic development.

## 1 Introduction

*SMARCA4*, which encodes the transcription activator, BRG1, is a component gene of the SWI/SNF (SWItch/Sucrose NonFermentable) chromatin remodeling complex; undifferentiated tumors associated with its functional deletion have been described in several organs [1, 2]. At present, no standard treatment strategy exists for this type of tumor. Even when complete surgical resection is achieved, many cases recur soon after surgery [1]. Chemotherapy is a common treatment option for unresectable or metastatic cases, but its efficacy is generally poor [3]. Recently, the enhancer of zeste homolog 2 (EZH2) inhibition showed a meaningful antitumor activity against SWI/SNF deficient tumors, and Tazemistat, an EZH2 inhibitor, is now being tested in early clinical trials [4]. Another treatment approach for this type of tumor is immunotherapy. Despite several reports suggesting that immune checkpoint inhibitors (ICIs) have potential as therapeutic options for SMARCA4‐deficient tumors [5], no established treatment for these tumors currently exists. In this study, we report a case of a SMARCA4‐deficient undifferentiated urothelial carcinoma that was effectively treated with nivolumab (Nivo; anti‐programmed cell death protein [PD‐1] blockade) after early relapse following treatment for non‐invasive bladder cancer.

## 2 Case Presentation

**FIGURE 1:**
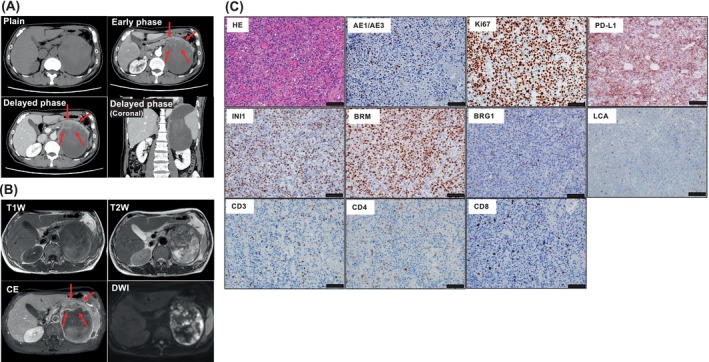
Radiological and histopathological findings of a retroperitoneal tumor. (A and B) The upper‐left panel shows the multiphase‐enhanced CT, and the lower‐left panel shows images obtained by Gd‐enhanced MRI before tumor excision. Red arrows indicate the solid component of the tumor. (C) The right panel shows the histopathologic features of a SMARCA4‐deficient retroperitoneal tumor. The solid tumor consisted of undifferentiated tumor cells focally showing a rhabdoid feature. On immunohistochemistry, the tumor cells were positive for SMARCB1 (INI1) and SMARCA2 (BRM) but negative for SMARCA4 (BRG1). Scale bars = 100 μm. CE, contrast‐enhanced; CT, computed tomography; DWI, diffusion‐weighted imaging; Gd, gadolinium; HE, hematoxylin and eosin; MRI, magnetic resonance imaging; T1W, T1 weighted imaging; T2W, T2 weighted imaging.

In 202X, A 60‐year‐old man underwent a transurethral resection of a bladder tumor (TUR‐BT) for non‐invasive bladder cancer in the muscle layer, in Japan. Eight months after the TUR‐BT, he presented to our institution with persistent abdominal pain. An enhanced computed tomography (CT) scan revealed a left retroperitoneal 160 × 120 × 90 mm tumor. The tumor showed a large cystic lesion with a heterogeneously‐enhancing solid component that was located on the dorsal side of the pancreatic tail (Figure 1A,B). Although radiological findings did not lead to a definitive diagnosis, the rapid growth of the tumor suggested it had malignant potential. Therefore, a total tumor excision was performed. We performed the retroperitoneal tumor resection by thoracoabdominal approach. Histologically, the resected tumor showed a partial rhabdoid morphology with substantial cell growth. Immunostaining revealed that BRG1 (SMARCA4) expression was lost in the tumor cells, whereas INI1 (SMARCB1) and BRM (SMARCA2) proteins were retained (Figure 1C). From these pathological observations, we initially diagnosed the patient with a SMARCA4‐deficient undifferentiated carcinoma of unknown primary (CUP). The tumor also displayed high Ki‐67 expression (80%) suggestive of aggressive behavior, and high PD‐L1 (80%). No noticeable infiltration of lymphocytes was found in the tumor microenvironment. Early postoperative local recurrence occurred 1 month after surgery in the form of a 150 × 144 × 95 mm retroperitoneal mass with a large cystic lesion and solid component. At the time of recurrence, the patient was malnourished with chronic inflammation (total protein: TP 6.1 g/dL, albumin: Alb 2.8 g/dL, white blood cells: WBC 24.2 × 10^3^/μL, C‐reactive protein: CRP 7.2 mg/dL) and a poor Eastern Cooperative Oncology Group Performance Status (PS 3) due to rapid progression of the tumor. Because of the severity of the patient's clinical condition, the administration of combination therapy with cytotoxic anticancer agents was inadequate for this patient. Therefore, he was urgently started on systemic therapy with Nivo monotherapy (240 mg, every 2 weeks) for a CUP [6].

**FIGURE 2:**
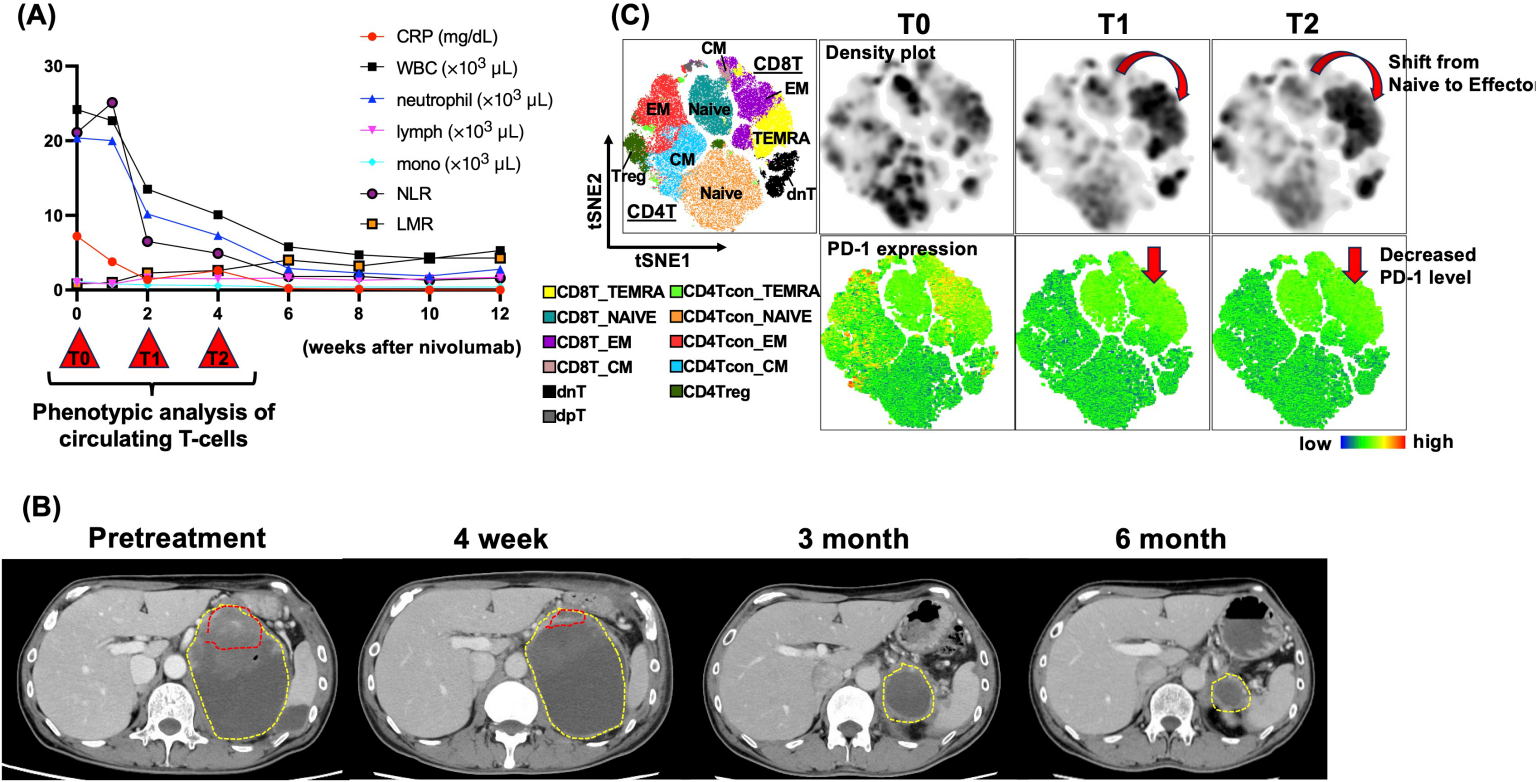
Treatment course after starting nivolumab. (A) The X‐axis shows the levels of CRP, WBC, neutrophil count, lymphocyte count, monocyte count, NLR, and LMR. (B) Red dashed lines on CT images highlight the solid component of the tumor, and yellow dashed lines indicate the cystic component. (C) Phenotypic and functional changes in peripheral T‐cells after nivolumab. Flow cytometry‐based immune phenotyping was performed using peripheral blood samples collected on day 0 (T0), day 15 (T1), and day 29 (T2) after nivolumab therapy; three health donor PBMCs were also used as reference controls. The left panel shows a viSNE plot with cells colored according to each cell population; each small dot represented a single cell. A total of 175 871 cells were used to generate the viSNE plot. Based on unsupervised cluster analysis of immune cell types and checkpoint expression, the accumulation of TEMRA CD8+T cells was observed (density plot). A comparison of PD‐1 expression on T cells at T0/T1/T2 showed reduced PD‐1 expression after nivolumab treatment (PD‐1 expression plot). CT, computed tomography; CRP, C‐reactive protein; LMR, lymphocyte‐to‐monocyte ratio; lymph, absolute neutrophil count; mono, absolute monocyte count; neutrophil, absolute neutrophil count; NLR, neutrophil‐to‐lymphocyte ratio; WBC, white blood cell count. CD4T (CD3+CD4+); CD8T (CD3+CD8+); CD4Treg (regulatory CD4T, CD3+CD4+CD25+CD127−); CM (central memory, CD45RA−CCR7+); dnT (double negative T, CD3+CD4−CD8−); EM (effector memory, CD45RA−CCR7−); Naive (CD45RA+CCR7+); PBMCs, peripheral blood mononuclear cells; PD‐1, programmed cell death protein 1; TEMRA (terminally differentiated effector memory cells re‐expressing CD45RA, CD45+CCR7−); tSNE, t‐distributed stochastic neighbor embedding; ViSNE, visualization of tSNE.

Just 2 weeks after starting Nivo, the patient's abdominal pain decreased, and his general condition also dramatically improved (PS 0), with an ameliorated nutritional status and inflammation markers (TP 7.0 g/dL, Alb 3.5 g/dL, WBC 13.5 × 10^3^/μL, CRP 1.4 mg/dL). The absolute lymphocyte count increased gradually with Nivo treatment, and the lymphocyte‐to‐monocyte ratio (LMR) and neutrophil‐to‐lymphocyte ratio (NLR), which reflect and/or predict the effect of ICI [7], also showed improvement. After 1 month of Nivo administration, CT revealed marked tumor shrinkage. Tumor shrinkage was sustained thereafter, and the entire tumor, including the cystic component, had markedly shrunk to 31 mm in size, with the nodular component completely disappearing 6 months after Nivo therapy (Figure 2A,B). At the time of writing, the patient maintains a durable partial response without any evidence of tumor regrowth for more than 10 months since the initial infusion of Nivo.

**FIGURE 3:**
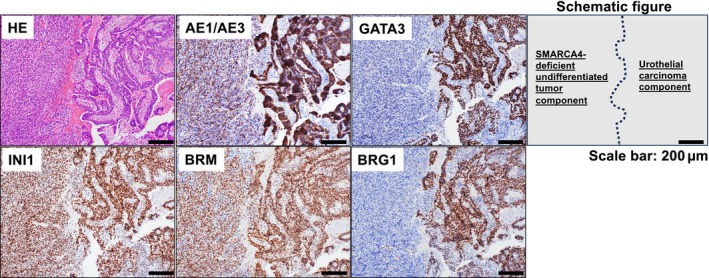
Histopathologic features of previous bladder tumor. The left side of each image shows the SMARCA4‐deficient undifferentiated tumor component and the right side shows the urothelial carcinoma component. Immunostain results of the SMARCA4‐deficient undifferentiated tumor component: AE1/AE3 (focal+), GATA3 (−), INI1 (+), BRM (+), and BRG1 (−). Immunostain results of the adjacent urothelial carcinoma component: AE1/AE3 (+), GATA3 (+), INI1 (+), BRM (+), and BRG1 (+). Scale bars = 200 μm. HE, hematoxylin and eosin.

A review of previous TUR‐BT specimens from the same patient indicated that a previous bladder tumor mainly consisted of a SMARCA4‐deficient undifferentiated tumor component (GATA3 [−], BRG1 [−]) with an adjacent urothelial carcinoma component (GATA3 [+], BRG1 [+]; Figure 3). From these pathological observations, it is suggested that an undifferentiated tumor component with a SMARCA4 deficiency arose, in part, from a background of preexisting urothelial carcinoma, resulting in early retroperitoneal recurrence after TUR‐BT. Therefore, the patient was finally diagnosed with a retroperitoneal recurrence of a SMARCA4‐deficient undifferentiated urothelial carcinoma.

Comprehensive genomic profiling (CGP) using an OncoGuide NCC Oncopanel System [8] was also performed on the retroperitoneal tumor. Five clinically relevant alterations were detected by genomic profiling (Table S1). Of those, SMARCA4 c.3216‐2A>G splicing variant was not yet registered in COSMIC v.98 (https://cancer.sanger.ac.uk/) or ClinVar (https://www.ncbi.nlm.nih.gov/clinvar/) databases but was expected to be pathogenic, showing high dbscSNV scores of 1 (ADA) and 0.924 (RF; cut‐off value: 0.6) [9]. The variant allele frequency (VAF) of this *SMARCA4* splicing variant was 42%, which was higher than the VAFs of other detected genetic variants (about 20%). These observations suggested that the tumor had a *SMARCA4* c.3216‐2A>G splicing variant and concomitant loss of heterozygosity (LOH) or copy‐neutral LOH, as previously reported in other SMARCA4‐deficient tumors [10]. In addition, we observed that the tumor mutational burden (TMB) was high (11.6 mt/Mb). These CGP results further support the pathological diagnosis of a SMARCA4‐deficient tumor.

## 3 Phenotypic Analysis of Peripheral Blood Immune Cells

To capture the ameliorated immune microenvironment, we next conducted a phenotypic analysis of peripheral blood lymphocytes using multi‐color flow cytometry (Table S2). As a result, we observed a reduction of naive CD8+T cells (T0; 39.2%, T1; 19.2%, and T2; 17.3% of total CD8+T cells) and the induction of terminally differentiated effector memory CD8+T cells (Figure 2C). These phenotypic changes in circulating T cells further support the findings that tumor‐specific cytotoxic T cells were activated by Nivo, leading to a dramatic anti‐tumor response in this patient.

## 4 Discussion

At the time of writing, no clear recommended treatment existed for SWI/SNF‐deficient urothelial carcinomas; the effect of ICIs on SWI/SNF‐deficient tumors is still considered to be controversial [5]. On one hand, several reports showed the promising antitumor activity of ICIs against this type of tumor [11, 12, 13, 14], on the other hand, others reported the dessert immune microenvironment of SMARCA4‐deficient tumors with limited efficacy to ICIs [15]. In our present case, a marked anti‐tumor effect of ICI was demonstrated, and non‐biased T‐cell clustering using multi‐color flow cytometry clearly supported an altered antitumor immune response. We observed a high level of PD‐L1 expression on the recurrent tumor (80%), which may have enhanced the efficacy of Nivo. Additionally, from the CGP test, we also observed high TMB (11.6 mt/Mb) in this case, which potentially increases the formation and presentation of immune neoantigens, thereby inducing an effective anti‐tumor immune response [16]. The efficacy of cytotoxic chemotherapeutic agents against SMARCA4‐dUT is limited. Therefore, the use of ICIs preferentially over chemotherapy may be beneficial, especially for patients with SWI/SNF‐deficient tumors with TMB‐high and high PD‐L1, as in this case. In lung cancer, SWI/SNF deficiency may lead to increased PD‐L1 expression and a high TMB; however, the molecular background is unknown and needs further evaluation [17].

In this present case, the histological morphology of the rhabdoid‐like undifferentiated tumor led us to suspect a SWI/SNF‐deficient tumor, and subsequent immunostaining led to the diagnosis of a SMARCA4‐deficient undifferentiated tumor. This effort also led to the identification of the developmental origin of this SMARCA4‐deficient undifferentiated tumor as a non‐invasive bladder cancer. It is possible that SWI/SNF‐deficient tumors are overlooked in a certain number of cases since SWI/SNF‐related proteins are not routinely immunostained in various types of cancer.

## 5 Conclusion

Regardless of the organ of cancer origin or cancer type, SWI/SNF‐deficient tumors should be suspected in undifferentiated and dedifferentiated tumors, and ICI may be considered as a promising treatment option for this type of tumor. The pathogenesis of SMARCA4‐deficient anaplastic tumors awaits further elucidation for therapeutic development.

## Author Contributions

**Yohei Arihara:** conceptualization, investigation, visualization, writing–review and editing, writing–original draft, resources. **Ginji Omori:** visualization, writing–original draft. **Ko Kobayashi:** resources, supervision. **Shintaro Sugita:** investigation, data curation, resources, supervision. **Kazuyuki Murase:** resources. **Tomohiro Kubo:** investigation, data curation. **Masashi Idogawa:** investigation, supervision, data curation. **Tadashi Hasegawa:** supervision. **Kohichi Takada:** supervision, writing–review and editing, conceptualization.

## Ethics Statement

Written informed consent was obtained from the patient to be published in this case report.

## Conflicts of Interest

K.T. received lecture fees from Daiichi Sankyo Co. Ltd., Eisai Co. Ltd., Janssen, Chugai Pharmaceutical Co. Ltd., and Sysmex Corporation. The remaining authors declare that they have no known competing financial interests that might affect the research reported in this article.

## Supporting information


**Table S1.** Summary of comprehensive genomic profiling test.
**Table S2.** List of antibodies and a brief method for the flow cytometry analysis.

## Data Availability

The data that support the findings of this study are available from the corresponding author upon reasonable request.
