# Supplementary material for: Marked Response to Nivolumab by a Patient With SMARCA4‐Deficient Undifferentiated Urothelial Carcinoma Showing High PD‐L1 Expression: A Case Report
Source: Cancer Rep (Hoboken). 2024 Jun 24;7(6):e2127. doi: 10.1002/cnr2.2127 (PMC11194675; doi:10.1002/cnr2.2127)
Supplement: Supplementary file 1 — Table S1. Summary of comprehensive genomic profiling test. Table S2. List of antibodies and a brief method for the flow cytometry analysis. [file CNR2-7-e2127-s001.docx]

**Supplemental Table S1.**

**Summary of comprehensive genomic profiling test**

| Gene name | Mutation type | CDS | Amino acid | VAF |
| --- | --- | --- | --- | --- |
| *SMARCA4* | splicing | Exon24:c.3216-2A>G | - | 42.0% |
| *PIK3CA* | nonsynonymous SNV | Exon10:c.1633G>C | E545Q | 17.9% |
| *HRAS* | nonsynonymous SNV | Exon2:c.37G>C | G13R | 45.8% |
| *CREBBP* | nonsynonymous SNV | Exon7:c.1642G>A | E548K | 22.0% |
| *EP300* | nonsynonymous SNV | Exon21:c.3724G>C | E1242Q | 22.0% |
| MSI status: stable | |  |  |  |
| TMB: TMB-High (11.6mt/Mb) | |  |  |  |

Footnote of this table:

CDS, CoDing Sequence; MSI, microsatellite instability; VAF, variant allele frequency, TMB, tumor mutational burden.

**Supplemental Table S2.**

**List of antibodies and a brief method for the flow cytometry analysis**

| **Antibody** | **Fluorochrome** | **Clone** | **Vendor** | **Catalog #** |
| --- | --- | --- | --- | --- |
| CD3 | APC-Cy7 | SK7 | BioLegend | 344818 |
| CD4 | BV510 | RPA-T4 | BioLegend | 300546 |
| CD8 | PE-Cy7 | SK1 | BioLegend | 344712 |
| CD45RA | PerCP | HL100 | BioLegend | 304156 |
| CCR7 | BV421 | G043-H7 | BioLegend | 353208 |
| CD25 | FITC | m-A251 | BioLegend | 356106 |
| CD127 | APC | A019D5 | BioLegend | 351316 |
| PD-1 | PE | A17188A | BioLegend | 379210 |
|  |  |  |  |  |

Peripheral blood mononuclear cells (PBMCs) were collected at several time points during Nivo treatment (T0; day 0, T1; day 15, and T2; day 29). Subsequently, cryopreserved PBMCs were stained with a panel of directly conjugated monoclonal antibodies to define each cell subset. After staining, cells were acquired using a BD FACS Canto II flow cytometer (BD, San Jose, CA, USA) and analyzed using FlowJo software (v.10.9.0, BD). To interpret high-dimensional multi-color flow cytometry data, a t-distributed stochastic neighbor embedding algorithm was performed.
